# Template Synthesis (Self-Assembly) of Macrocycles: Theory and Practice

**DOI:** 10.3390/molecules27154829

**Published:** 2022-07-28

**Authors:** Oleg V. Mikhailov

**Affiliations:** Department of Analytical Chemistry, Certification and Quality Management, Kazan National Research Technological University, K. Marx Street 68, 420015 Kazan, Russia; ovm@kstu.ru or olegmkhlv@gmail.com

For more than 60 years, in coordination chemistry (and since the beginning of the 21st century, in molecular nanotechnology, too), there has been very significant interest in template synthesis reactions, in which the design of coordination compounds (metal complexes) with complex ligands is carried out not according to the classical scheme [metal ion + ligand → complex], but according to scheme [metal ion + “building blocks” of the future ligand (the so-called ligand synthons or ligsons) → complex]. Generally, template synthesis in coordination chemistry refers to such complex-formation reactions in which a metal ion (or other reaction center) with a certain stereochemistry and electronic state (the so-called template) acts as a kind of template for the formation of the corresponding starting substances (ligsons), the only possible or predominant chemicals under the reaction conditions, the synthesis of which in the absence of this very template is either difficult or cannot be realized at all. Template synthesis can be considered a special case of so-called “self-assembly”, which has long been used in organic chemistry for the synthesis of very complex organic compounds from rather simple substances; however, generally, “self-assembly” does not necessarily imply the presence in the reaction system of any special agent that directs the reaction in a certain direction. The metal ion thus plays the role of a type of “conductor” (“kapellmeister”) that controls the process of “self-assembly”; in its absence, by definition, the process of template synthesis does not occur at all. For example, the reaction between nickel(II) chloride, 2-hydroxybenzaldehyde and ammonia proceeding according to the general Equation (1)

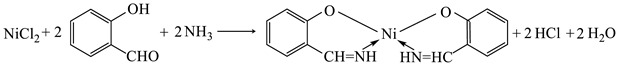
(1)
is a template synthesis, since the reaction between 2-hydroxybenzaldehyde and ammonia with the formation of 1-hydroxy-2-iminomethylbenzene C_6_H_4_(OH)(CH=NH) in the absence of the Ni(II) ion does not occur, while the reaction (2), which is close to it, in which any alkylamine NH_2_R is used instead of ammonia,

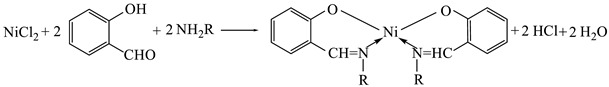
(2)
is not a template synthesis, because the reaction of the formation of a chelate ligand (3) that forms a complex with Ni(II), and namely

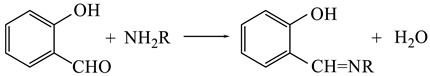
(3)
proceeds both in the presence and in the absence of this metal ion. “Self-assembly” with the participation of various templates, in particular, underlies the synthesis of many so-called metal macroheterocyclic compounds, the importance of which, both for fundamental science and in practical terms, has clearly increased in recent years. Reactions of “self-assembly” also currently occupy a dominant position in the synthesis of porphyrins, crown ethers, cryptands, rotaxanes and other macrocyclic systems with closed contours containing various heteroatoms; such compounds are often obtained from the metal macroheterocyclic compounds formed during the template synthesis, by their demetallation. The final products of these reactions, having non-trivial physical and chemical properties, have extremely diverse applications; in addition to chemistry proper, they are used in metallurgy and medicine, industrial biotechnology and catalysis, microelectronics, agriculture, and many other fields.

It is generally accepted that, for the first template, synthesis was performed by the chemist N. Curtis (New Zealand) in the pioneer work [1]. In particular, in this work, a “nickel-mediated” condensation of tris(ethylenediamine)nickel(II) tetraoxochlorate(VII) [Ni(H_2_NCH_2_CH_2_NH_2_)_3_](ClO_4_)_2_ with acetone (4) that allowed obtaining Ni(II) complexes with a macroheterocyclic ligand derived from 1,4,8,11-tetraazacyclotetradecadiene was found. As a result, a mixture of two macrocyclic compounds was formed, in the first of which the nitrogen atoms bound by double bonds with carbon atoms are in cis-positions; in the second, they are in trans-positions relative to each other.

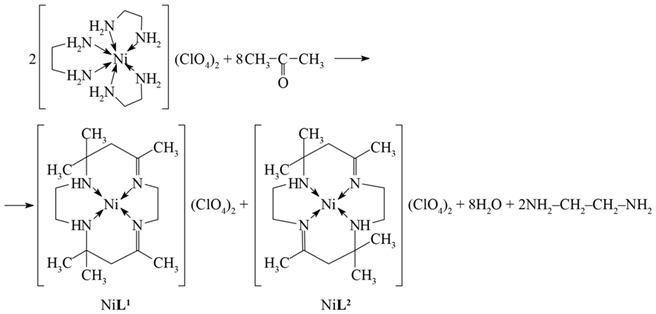
(4)

However, it should be noted that, more than thirty years before the publication of the above article, namely, in 1927, the template process was *completely accidentally* carried out by Japanese chemists due to the reaction of 1,2-dibromobenzene and copper (I) cyanide in pyridine, because, instead of the expected colorless target product, benzenedicarbonitrile-1,2 (1,2-dicyanobenzene), a dark blue substance, was formed, which, many years later, was identified as copper(II) phthalocyanine [2], which is now one of the best known macrocyclic compounds (Figure 1). Remarkably and even symptomatically, in the period between these two events, no reports of template processes were noted in the literature. Nevertheless, a huge number of similar macrocyclic compounds were subsequently synthesized (now having reached at least a five-digit number), so this variant of complex formation had already been sufficiently well studied by the beginning of the 21st century [3,4,5,6].

Template processes have a number of outwardly similar (and sometimes even coinciding) features compared to well-known processes in coordination chemistry such as direct complexing, reactions of coordinated ligands (reactions “in situ”), metal removal (“demetallation”), transmetallation, and catalytic reactions. This sometimes leads to the unreasonable attribution of such reactions to template processes. The place of template reactions among the entire array of listed chemical processes can be reflected in the following scheme (Figure 2):

In the truly vast literature devoted to the template synthesis of metal macroheterocyclic compounds, a number of specific terms and expressions are used, the most important of which are the following [6,7,8,9,10,11]:

*Template center* (a template, matrix, mold, form, model, or pattern)—a metal ion or another particle (molecule, ion, or radical) that can orientate and activate the ligands for their subsequent interaction.

*Positive template*—a template that brings together the reactive end groups of a molecule (molecules), facilitating intramolecular coupling.

*Negative template*—a template that prevents the reactive end groups of a molecule (molecules) coming together, suppressing intramolecular coupling and favoring in-termolecular reactions. 

*Template bonds*—forces by means of which the corresponding template orients (and/or activates) the reacting ligands, preparing them for the reaction. Metal–ligand binding, hydrogen bonding, and π−π interactions can be successfully exploited with a high degree of control in syntheses of macrocycles.

*Ligand synthon* (abbreviated: *ligson*)—a polyfunctional, usually chelating, ligand that participates in the assemblage reactions at the template center (a “building block” for the template synthesis).

*Ligand product* (*chelant*)—a ligand that is a product formed as a result of ligson interactions and that occupies several coordination places in the inner coordination sphere of the macroheterocyclic compound formed as a result of this process.

*Template information*—the totality of the coordinative-stereochemical characteristics of the template center that stipulates a definite spatial arrangement of ligsons.

*Template complementarity*—a matching between the template information of the matrix and the geometrical (conformational) and electron donor–acceptor parameters of the ligsons and of the ligand product.

Separately, the term *macrocycle*, which has an ambiguous interpretation, should be discussed. In organic chemistry, it is understood as any molecule containing a ring (a closed group of atoms) of more than nine atoms. According to the IUPAC definition, a macrocycle is a cyclic macromolecule or a macromolecular cyclic part of a macromolecule. However, in coordination chemistry, macrocycles include cyclic atomic groups with three or more donor atoms capable of forming coordination bonds with the central metal atom. However, it should be noted that the term “metal macroheterocyclic compound” is not currently very popular regarding the chemistry of macrocyclic compounds. Nevertheless, it seems to us that it is quite adequate to simply use SO to refer to any cyclic metal-containing chemical compound with nine or more atoms in the macrocycle, of which at least three are electron pair donors.

Currently, it is customary to subdivide macrocyclic metal complexes into two categories, with the so-called open contour and so-called closed contour. The first of these groups includes chelate complexes, in which the metal atom (complexator) is located on the *rim* of the macrocycle and is one of the atoms that form it; examples of this kind are complexes **A** and **B**. The second category includes chelate complexes, in which the complexing metal atom is located *inside* the macrocycle and is not among the atoms that form it; these include, for example, complexes **C** and **D** (Figure 3).

On the other hand, it is useful to systematize macrocyclic metal complexes on the basis of the total number of cyclic groups containing the complexing metal ion (the so-called metal-chelate cycles). In this regard, at least two more groups of these coordination compounds appear: macrotricyclic, containing three metal-chelate cycles, and macrotetracyclic, containing four metal-chelate cycles (although, in principle, mononuclear macrocyclic metal complexes with an even greater number of metal-chelate cycles are also possible). In this line, it should be noted that this second variant of the taxonomy of macrocyclic metal complexes is not dependent on the first variant, despite the fact that, as a rule, macrotricyclic complexes are open-contour complexes, while macrotetracyclic complexes are closed-contour ones (see, for example, the above-mentioned complexes **A** and **B**, and **C** and **D**, respectively); there may be closed-contour macrotricyclic metal complexes (for example, **E**) and open-contour macrotetracyclic ones (for example, **F**) (Figure 3). It is also advisable to introduce special symbols for metal– macroheterocyclic compounds, indicating for them, by numerical indexing, on the one hand, the total number of metal-chelate cycles, and, on the other hand, the total number of atoms contained in each of these cycles. The number of these digits (usually three or four, although more may be possible in principle) will indicate the total number of metal-chelate cycles in the given metal macroheterocyclic compound. In the numbering of metal-chelate cycles, it would be logical to start from the leftmost cycle and then move clockwise along the perimeter of the macrocycle (since spelling in most languages of the world is from left to right). Within the framework of this approach, the macrocyclic complex **A** indicated above, containing two 5-membered and one 7-membered cycle, should be denoted by the symbol (575); complex **B**, which contains three 6-membered cycles, by the symbol (666); complex **C**, by the symbol (5757); complex **D**, by the symbol (6586); complex **E**, by the symbol (656); complex **F**, by the symbol (5756).

In many cases, the process of the synthesis of metal macroheterocyclic compounds within the framework of “self-assembly” (albeit in a somewhat simplified form) can be represented as follows. In the first stage, the metal ion reacts with any of the “building blocks”, forming an intermediate chelate complex with this “building block”. In the second stage, another “building block” reacts with this intermediate formed in the first stage and performs a kind of “crosslinking” of two or more chelate rings of the complex formed in the first stage into a single cyclic contour (macrocycle). Therein, three variants are possible: (a) the preservation of all the metal–heteroatom bonds that were in the intermediate chelate complex; (b) the replacement of some of the metal–heteroatom bonds that were in the intermediate chelate complex with other metal–heteroatom bonds; (c) the complete replacement of the metal–heteroatom bonds present in the intermediate chelate complex with other metal–heteroatom bonds [8]. An example of the realization of the first of these variants is the reaction (4) described above for the formation of the (5656) macrotetracyclic metal complex, carried out by N. Curtis; an example of the realization of the second variant is the reaction (1) between NiCl_2_, 2-hydroxybenzaldehyde, and NH_3_; an example of the realization of the third variant is the reaction between CuCl_2_, pentanedione-2,4, and propanediamine-1,3.

As already mentioned at the beginning of our story, the metal ion acts as a “template” or “matrix” that directs the process in the “right direction” (hence, the reactions of the synthesis of metal macroheterocyclic compounds are often called “reactions on matrices”). There are, however, a number of factors, the presence of which is absolutely necessary for “candidates” for the role of a template center, namely, the correspondence of the radius of the internal cavity formed during the “assembly” of the chelant to the radius of the metal ion, the correspondence of the number of donor atoms inside this cavity to the coordination number characteristic for a given metal ion, a certain geometric orientation of the donor atoms in the cavity corresponding to the coordination polyhedron optimal for a given metal ion, and conformational flexibility that allows chelant donor atoms to move within the cavity with minimal energy expenditure so that the previous state can be fulfilled. These are, so to speak, “geometric constraints”; however, the electronic characteristics of the metal ion are also important, in particular, its ability to participate in π-dative interactions and corresponding effective charges. Nevertheless, occasionally, metal ions that are similar in “geometric” and “electronic” parameters, in terms of the active functions of the template, lead to very different directions of synthesis and, consequently, to the nature of the final products formed. Moreover, a metal ion, performing the function of a “template”, sometimes “leaves” the inner sphere of the macrocyclic complex formed by it—so-called demetallation occurs. Figuratively speaking, “Der Mohr hat seine Schuldigkeit getan, der Mohr kann gehen” (“The Moor has done his duty, the Moor can go.”).

Although, at present, the number of studies devoted to template synthesis is many thousands (if not tens of thousands), in this area of chemical synthesis, the problem of the structural–chemical design and control of this synthesis, as well as predicting the specifics of its products, remains. This problem clearly manifests itself during the “self-assembly” of the so-called small metallocycles, the results of which in each particular case are difficult to predict, unless any close analogs of such a process are known. Additionally, rather often, even ions similar in geometrical and electronic parameters, playing the role of a template, “organize” this “self-assembly” in completely different directions. Particularly interesting in the reactions of “self-assembly” are the so-called ambidentate, primarily (N,O,S,P)-donor atomic ligand synthons, which, depending on the starting conditions for the complexation and the nature of the metal ion, are capable of being coordinated to the latter by means of various donor atoms. The given circumstance, on the one hand, opens up new opportunities for the targeted synthesis of a wide variety of metal macrocyclic compounds and, on the other hand, in some cases, makes it possible to obtain new, previously unknown metal complexes; this is undoubtedly valuable for both fundamental and applied coordination and supramolecular chemistry (see the articles [12,13,14,15,16,17] for further information).

As a rule, template synthesis is accompanied by a decrease in the total entropy (*ΔS*) of the reaction system (sometimes quite significant). As a result, in accordance with the general Gibbs–Helmholtz equation for the isobaric process *ΔG =* *ΔH − TΔS*, with increasing temperature, *T*, the probability of this process must decrease. At low temperatures, this process practically does not flow due to its kinetic retardation; at relatively high temperatures, it is thermodynamically forbidden. In a number of cases, this unfavorable situation can be improved by combining a sufficiently high temperature with an increased pressure and carrying out the process for a rather long time (up to several hours). In this line, one of the promising variants for its realization is its implementation in so-called organizing media with a preliminary decrease in entropy (for example, in polymer-immobilized matrix systems, where template synthesis occurs in intermolecular cavities that can be considered peculiar molecular nanoreactors) [16,17,18,19]. In this variant, it becomes possible to quite noticeably decrease the difference between the entropy of the initial and final states of the reaction system and, as a result, to expand the temperature range at which *ΔG* has a negative value. It is also possible to reduce the energy barrier for process activation at the same time, which increases the probability of its implementation in experiments. In addition, it becomes possible to realize such reactions that are thermodynamically forbidden in solution or solid phase. The foregoing is especially important right now, when the efforts of the vast majority of chemists working in the field of coordination chemistry, all over the world, are primarily directed toward the development of new methods for the synthesis of metal macrocyclic and supramolecular compounds. In this line, we have to state with regret that, to date, there have been very few studies devoted to template synthesis in organizing media.

Taking into account all of the above, we hope that this Special Issue will make at least a small contribution to this specific and important branch of modern chemistry and will facilitate the emergence of further research in this direction.

## Figures and Tables

**Figure 1:**
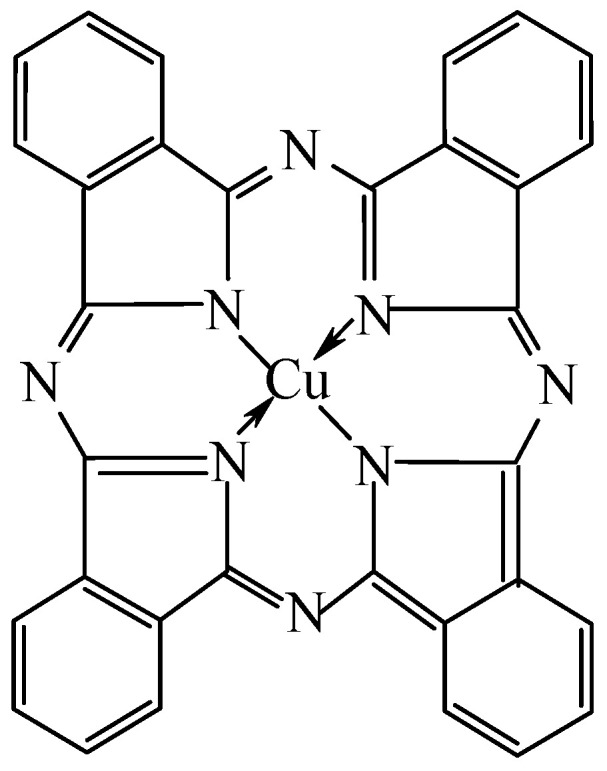
The structural formula of copper(II) phthalocyanine.

**Figure 2:**
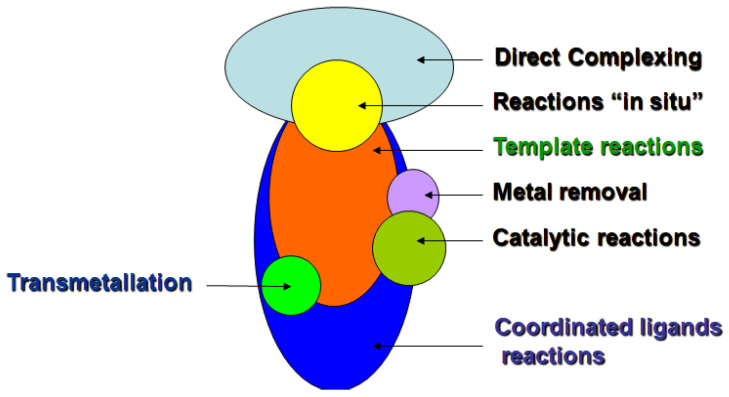
The place of template processes in modern coordination chemistry.

**Figure 3:**
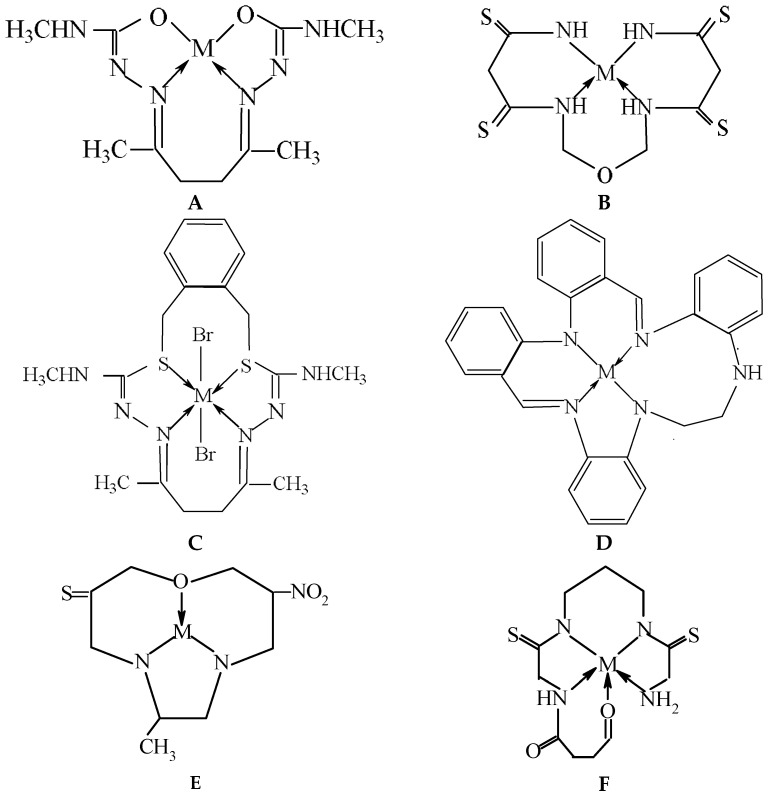
The examples of macrocyclic metal complexes with open contour (**A**,**B**,**F**) and closed contour (**C**–**E**).
